# Encased by Infection: A Rare Case of Retroperitoneal Fibrosis Induced by Disseminated Cysticercosis

**DOI:** 10.1155/crdi/6221398

**Published:** 2025-06-01

**Authors:** Lufeng Zhang, Shaoyang Zhan

**Affiliations:** Department of Urology, Hefei BOE Hospital, Hefei, Anhui, China

## Abstract

Cysticercosis refers to a disease caused by the larvae of *Taenia solium* parasitizing various tissues and organs of the human body. It is reported that cysticercosis is most commonly caused by the central nervous system, and retroperitoneal fibrosis is rare. We report a case of retroperitoneal fibrosis caused by cysticercosis and a urinary tract obstruction caused by retroperitoneal fibrosis, which was successfully relieved by transurethral stenting. The complex pathophysiological mechanism of cysticercosis-induced retroperitoneal fibrosis requires further study. In the diagnosis and treatment of the disease, it is very important to consider the patient's epidemiological history and the presence of parasite infection.

## 1. Introduction

Retroperitoneal fibrosis (RPF) is a rare disease whose histological features are inflammatory-fibrous tissue surrounding the abdominal aorta, iliac arteries, and even extending to the retroperitoneum and surrounding adjacent tissues such as ureter and inferior vena cava, resulting in a series of compression symptoms [1]. RPF is divided into primary (also called idiopathic) and secondary types according to the cause. The etiology of primary RPF is unknown, but some studies suggest that it is the result of autoimmune inflammation [2]. Secondary RPF has a clear etiology, mostly secondary to tumors, drugs, radiation therapy, etc. Among them, lymphoma, retroperitoneal sarcoma, carcinoid, and primary tumor metastasis are malignant causes of secondary RPF [3]. However, RPF caused by cysticercosis is rare and has not been reported at home and abroad. A case of retroperitoneal fibrosis induced by disseminated cysticercosis was admitted to our hospital.

## 2. Case Presentation

**Figure 1:**
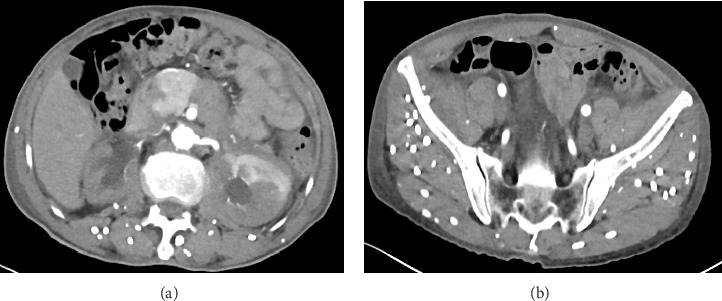
Abdominal CT scan showing (a) retroperitoneal fibrosis encasing the ureters, and (b) multiple calcified lesions consistent with cysticercosis.

**Figure 2:**
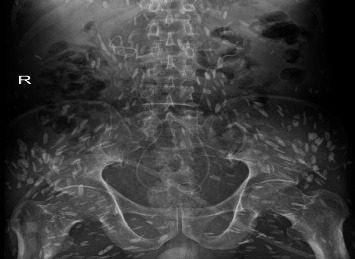
Kidney-Ureter-Bladder (KUB) radiograph, after ureteral stent placement.

The patient, male, 69 years old, BMI: 20.03 kg/m, was admitted to the hospital for more than 3 months due to low back pain and discomfort. Before admission, magnetic resonance urography (MRU) showed that both kidneys and the middle and upper ureters were dilated with hydrops, and RPF was possible. Renal function test showed: creatinine: 0.356 mmol/L, urea: 20.97 mmol/L. After admission, plain and enhanced abdominal and pelvic scans showed: (1) abnormal density around the abdominal aorta and bilateral ureters, considering the possibility of idiopathic RPF (Figure 1(a)), please combine clinical; (2) change of chronic pancreatitis; (3) abnormal enhancement of pancreas and left kidney parenchyma, consider abnormal perfusion, follow-up; (4) local abnormal density in venous phase of left internal lobe of liver, consider local poor perfusion; (5) right kidney atrophy; bilateral renal pelvis and bilateral The upper segment of the ureter was dilated with hydrops; (6) pleural effusions and ascites were present on both sides; (7) there were multiple short-strip high-density shadows in the subcutaneous and abdominal pelvic cavity of the chest, abdomen, and pelvis, and calcification of cysticercosis was considered (Figure 1(b)); (8) prostate calcification; (9) the right renal artery was slender, and calcified plaques formed at the origin of bilateral renal arteries and abdominal aorta. Renal function test showed: creatinine: 376.0 mmol/L, urea: 23.4 mmol/L, K^+^: 6.0 mmol/L. Ureteroscopy and bilateral ureteral stent placement were performed. Postoperative re-examination of Kidney-Ureter-Bladder (KUB) radiograph showed the presence of bilateral ureteral stents (Figure 2). The patient's creatinine was 331.2 mmol/L, creatinine was 25.1 mmol/L, and K^+^ was 4.0 mmol/L 3 days after surgery. The patient was followed up after discharge for 2 years, during which the double J tube was replaced every 6 months. The creatinine was close to 170 mmol/L, urea was close to 12.9 mmol/L, and K^+^ was normal.

## 3. Discussion

Cysticercosis is a disease caused by *Taenia solium* larva parasitism in various tissues and organs of the human body. *Taenia solium* adult parasite in the human intestine, eggs with human excrement out of the body, when the eggs are swallowed by the intermediate host (pig), can develop into cysticercus in the pig, when people eat raw pork containing cysticercus can cause tapeworm disease, also can cause cysticercus disease, also known as cysticercosis [4]. In 2010 and 2014, it was listed as one of the neglected tropical diseases and negligible zoonotic diseases by the World Health Organization (WHO) and the Food and Agriculture Organization of the United Nations (FAO), respectively [5]. Cysticercosis is spreading around the world and is a major health problem in most countries in Latin America, Africa and Asia. With globalization and increased exchanges between countries, more and more cases of cysticercosis are reported in non-endemic areas, and some developed countries have classified cysticercosis as an emerging infectious disease [4]. In China, the disease is mainly prevalent in the southwest region, especially in Yunnan Province, where a considerable number of residents have the habit of eating raw or undercooked pork. Cysticercosis can affect any tissue in the body, such as the central nervous system, subcutaneous tissue, eyes, and muscles [6]. The central nervous system is most common in clinical cases [7, 8].

Systemic disseminated cysticercosis is clinically rare, and the mechanism of retroperitoneal fibrosis in this patient is unclear. Parasites can produce a series of physical and chemical stimulation and inflammatory reactions in human body, such as mechanical stimulation and immune pathological damage caused by insect movement, and toxic reactions caused by insect secretions, excreta and corpses. In this case, it was considered that the formation of fibrous tissue caused by physical and chemical stimulation and inflammation caused by parasites might encase adjacent tissues, press the ureter and lead to ureteral obstruction, resulting in renal failure. Therefore, after the double J tube was indwelled, the creatinine and potassium decreased significantly and stabilized at a slightly higher normal value. Therefore, we recommend long-term indwelling of the double J tube to keep the ureter open.

The differential diagnosis for RPF includes idiopathic RPF, malignancies (e.g., lymphoma, retroperitoneal sarcoma), and infections (e.g., *tuberculosis*, fungal infections). Idiopathic RPF is typically associated with elevated IgG4 levels and responds to immunosuppressive therapy [9]. Malignancies often present with systemic symptoms (e.g., weight loss, night sweats) and show neoplastic cells on biopsy. Tuberculous RPF may manifest with caseating granulomas on histology and positive acid-fast bacilli staining.

In conclusion, systemic disseminated cysticercosis caused by *Taenia solium* infection is relatively rare in clinical practice, and the disease itself and the impact on the host are more complicated, which makes the diagnosis and treatment of clinical diseases more difficult. In clinical practice, the cause of parasites is often overlooked. In the diagnosis and treatment, the uncommon cause of parasites should be considered to avoid misdiagnosis and missed diagnosis, which will affect the diagnosis and treatment plan and prognosis of patients.

## Data Availability

All data generated or analysed during this study are included in this published article.

## Nomenclature

RPF: Retroperitoneal fibrosis
KUB: Kidney-Ureter-Bladder
MRU: Magnetic resonance urography
WHO: World Health Organization
FAO: Food and Agriculture Organization of the United Nations
